# Effectiveness of bazedoxifene in preventing glucocorticoid-induced bone loss in rheumatoid arthritis patients

**DOI:** 10.1186/s13075-021-02564-1

**Published:** 2021-07-02

**Authors:** Soo-Kyung Cho, Hyoungyoung Kim, Jiyoung Lee, Eunwoo Nam, Seunghun Lee, Yun Young Choi, Yoon-Kyoung Sung

**Affiliations:** 1 Hanyang University Hospital for Rheumatic Diseases, 222-1 wangsimni-ro, Seongdong-gu, Seoul, 04763 South Korea; 2 Clinical Research Center for Rheumatoid Arthritis (CRCRA), 222 wangsimni-ro, Seongdong-gu, Seoul, 04763 South Korea; 3 Department of Radiology, Hanyang University College of Medicine, 222 wangsimni-ro, Seongdong-gu, Seoul, 04763 South Korea; 4 Department of Nuclear Medicine, Hanyang University College of Medicine, 222 wangsimni-ro, Seongdong-gu, Seoul, 04763 South Korea

**Keywords:** Bazedoxifene, Rheumatoid arthritis, Glucocorticoids, Osteopenia, Bone loss

## Abstract

**Objective:**

To evaluate the effectiveness of bazedoxifene in preventing bone loss in patients with rheumatoid arthritis (RA) receiving low-dose glucocorticoids (GCs).

**Methods:**

In this randomized, controlled, open-label study, we assigned postmenopausal women with osteopenia who had been receiving low-dose GCs for RA to two groups: a group receiving bazedoxifene (20 mg/day) with elemental calcium 1200 mg and vitamin D 800 IU daily (bazedoxifene group) and a group receiving the same doses of calcium and vitamin D only (control group). As primary outcome, bone mineral density (BMD) change in the lumbar spine (L-spine) from baseline to 48 weeks was assessed. Changes in BMD in the femur, trabecular bone score, bone turnover markers, and development of fracture were assessed as secondary outcomes. For intention-to-treat analysis, 20 completed data sets were created by applying multiple imputations by chained equations.

**Results:**

A total of 114 patients (57 patients in each group) were recruited. A significant increase in L-spine BMD (0.015 g/cm^2^, *P* = 0.007) was observed in the bazedoxifene group, and the increase was significantly higher than in the control group (0.013, 95% CI 0.0003–0.026, *P* = 0.047). Reductions in bone turnover markers in the bazedoxifene group were significantly greater than in the control group. Only one fracture was observed in the bazedoxifene group, while four fractures developed in the control group.

**Conclusion:**

In postmenopausal patients with RA receiving low-dose GCs, bazedoxifene improved BMD and reduced bone turnover markers. However, the change in BMD did not exceed the least significant change.

**Trial registration:**

ClinicalTrials.gov, NCT02602704.

**Supplementary Information:**

The online version contains supplementary material available at 10.1186/s13075-021-02564-1.

## Introduction

Rheumatoid arthritis (RA), a chronic inflammatory arthritis, is associated with increased risk of osteoporosis and fracture [1, 2]. Inflammation and osteoporosis have been shown to be associated with one another in previous studies [3]. The release of pro-inflammatory cytokines such as interleukin (IL)-1, IL-6, and tumor necrosis factor-α (TNF-α) may increase osteoclast activation, thus disrupting the equilibrium between bone resorption and bone formation [4, 5]. There are multiple risk factors for osteoporosis other than inflammation in RA patients, including old age, female sex, menopause, and decreased physical activity [6]. The incidence of RA peaks in female patients aged 50–60 years who are postmenopausal [7], and glucocorticoid (GC) is frequently prescribed in early stage RA to reduce inflammation. Therefore, postmenopausal women with RA maybe at increased risk for bone loss and fracture.

The American College of Rheumatology guidelines for the prevention and treatment of glucocorticoid-induced osteoporosis (GIOP) recommend that all patients taking more than 2.5 mg/day prednisolone or equivalent for more than 3 months need to take adequate calcium and vitamin D [8]. Osteoporotic medications are recommended for adults over 40 who have a moderate or high risk of fracture based on the fracture risk assessment tool (FRAX) or a bone mineral density (BMD) T score ≤ − 2.5 for the hip or spine [8, 9]. Oral bisphosphonates as first line therapy have been shown to be effective in preventing fractures, when given with adequate calcium and vitamin D supplementation. However, long-term use of bisphosphonates is associated with increased risk of adverse events (AEs) such as jaw necrosis and atypical femur fracture [10]. In addition, many osteoporotic fractures occur in patients with osteopenia due to the fact that even though the risk of fracture is lower in osteopenia than in osteoporosis, the number of subjects at risk is much higher in the osteopenic range due to the Gaussian distribution of BMD values in the population [11]. Therefore, it is crucial to prevent osteopenia progressing to osteoporosis in patients receiving long-term GC therapy.

Selective estrogen receptor modulators (SERMs) bind to estrogen receptors but exhibit different selectivities in their estrogenic actions in different tissues. They prevent bone loss and lower the risk of fracture by modulating osteoblast and osteoclast activities to decrease bone turnover [12]. However, little is known about the efficacy of SERMs in GIOP. One controlled trial studied the efficacy of raloxifene on BMD in postmenopausal women with rheumatic diseases, who were chronic users of GC. This study demonstrated that raloxifene is an option for the prevention of BMD loss in postmenopausal women receiving long-term GC treatment [13]. Bazedoxifene is a third-generation SERM, and a 7-year randomized controlled trial (RCT) extension study and meta-analysis demonstrated that it is another good choice for preventing osteoporotic facture with more tissue selectivity than other SERMs [14–16]. However, there is little evidence regarding the effectiveness of bazedoxifene in preventing GIOP. This study aimed to evaluate the efficacy of bazedoxifene in preventing bone loss and fractures in RA patients with osteopenia on long-term GC therapy.

## Methods

### Study design

This was a randomized, controlled, open-label study conducted for 56 weeks. Four trial visits occurred over the course of the 56 weeks. At study entry, all patients who took elemental calcium (1200 mg daily) and vitamin D (800 IU daily) were assigned by blocks of two to receive either bazedoxifene (20 mg/day) (bazedoxifene group) or none (control group). Randomization was performed by an independent coordinator. Participants were followed-up at 24 and 48 weeks with special attention to RA flares and occurrence of AEs. Demographic characteristics such as age, sex, and medications related to RA, as well as laboratory results such as complete blood count (CBC), chemistry, and levels of inflammatory markers were collected at enrollment. BMD and trabecular bone score (TBS) were assessed at 0 and 48 weeks, and levels of bone turnover markers were assessed at 0, 24, and 48 weeks. At 56 weeks, the occurrence of AEs was assessed. The study design is shown in Additional file 1. Written informed consent was obtained from all patients before the performance of any trial procedures.

### Patient and public involvement

Patients were not involved in the design of this study.

### Study population

Postmenopausal women with osteopenia who had been receiving low-dose GCs for RA in the outpatient clinic of one university hospital were eligible for enrollment in this study. Inclusion criteria were as follows: (1) female RA patients ≥ 45 years old, self-reported to be postmenopausal for ≥ 12 months; (2) on low-dose glucocorticoids (prednisone ≤ 7.5 mg/day or equivalent) for ≥ 3 months prior to entry; (3) patients expected to be on GC treatment for 3 months after entry; and (4) mean T score of BMD between − 1 and − 2.5 in the L-spine or femoral neck. Exclusion criteria were as follows: (1) patients with two or more vertebral fractures (L1-L4), osteomalacia, renal osteodystrophy, hyperparathyroidism, severe renal impairment, or creatinine clearance < 30 ml/min; (2) history of deep vein thrombosis, pulmonary embolism, undiagnosed uterine bleeding, allergic reactions, or intolerance to SERMs; and (3) received osteoporosis medication including bisphosphonates, parathyroid hormone, SERMs, or anticonvulsants therapies within 6 months prior to entry.

### Outcomes of interest

BMD change in the L-spine from baseline to 48 weeks was assessed as the primary outcome measure. Secondary outcomes included changes in femur neck BMD, L-spine TBS, and bone turnover markers from baseline to 48 weeks, as well as the frequency of fractures.

#### BMD assessment

BMD of the L-spine (L1–4) and femur neck was assessed by dual-energy x-ray absorptiometry (DXA) (Hologic®, Discovery W, Hologic APEX software version 2.3.1; Bedford, MA, USA) using reference data from the Asian (Japanese) population. BMD in the L-spine was estimated as the mean of individual measurements for L1–L4 excluding any fractured or otherwise deformed vertebrae. The technician who was responsible for measuring BMD was blinded to the details of the study. In patients with a single spine fracture, BMD was calculated after excluding the values of involved vertebrae. The least significant change (LSC) of BMD was 0.024 g/cm^2^.

#### TBS assessment

Lumbar spine TBS was obtained using the spine DXA scan archived from the test at baseline and 48 weeks. It was calculated after reanalysis of the DXA scan of the L-spine using TBS iNsight® software (Version 2.0.0.1, Med-Imaps, Bordeaux, France). Vertebrae excluded in the calculation of BMD were also excluded in the TBS calculation.

#### Assessment of bone turnover markers

Serum levels of bone formation markers (serum bone-specific alkaline phosphatase and osteocalcin) and resorption markers (serum C-telopeptide and urine N-telopeptide) were assessed at 0, 24, and 48 weeks. Serum C-telopeptide were assayed by electrochemiluminescence (Roche Diagnostics, GmbH, Mannheim, Germany) and urine N-telopeptide was measured by chemiluminescence (Ortho Clinical Diagnostics, New York, USA) using commercially available kits. Serum osteocalcin and bone-specific alkaline phosphatase (ALP) levels were also determined by electrochemiluminescence using commercial kits (Beckman Coulter Inc., Brea, USA). All blood and urine samples were collected after at least an 8-h fast.

#### Fracture assessment

Baseline vertebral fracture was defined as a loss of at least 25% of vertebral height. Incident vertebral fractures at 48 weeks were diagnosed when there were distinct alterations in the morphology of the vertebral bodies that resulted in the loss of at least 25% of vertebral height of previously normal vertebrae. In addition, all subjects enrolled in this study reported the development of fractures up to 56 weeks.

#### Safety

All subjects who received a dose of the study drug were evaluated for safety for 56 weeks. Safety assessments included AEs and clinically significant changes in laboratory test results. The investigator classified the severity of each adverse event as mild, moderate, severe, or very severe. Radiographs of the thoracic and lumbar vertebrae were examined for deformities by a radiologist.

### Statistical analysis

Continuous data are presented as mean ± SD and categorical data by frequency (%). Analyses of primary and secondary outcomes were performed in the intention-to-treat population including all participants assigned to the bazedoxifene and control groups. Multiple imputations by chained equations (MICE) [17, 18] hiring the classification and regression tree (CART) [19] were applied to impute missing data. Since data for nine (15.8%) patients in the bazedoxifene group and six (10.5%) in the control group were missing in the week 48 observation, twenty imputed data sets were created [18]. A per-protocol analysis of the participants completed the study, as the protocol was implemented. Subgroup analysis was performed for the patients at high risk of fracture as identified by a 10-year major osteoporotic fracture probability exceeding 20%, or hip fracture exceeding 3% based on the FRAX tool.

After testing the significance of BMD change at the L-spine (primary outcome) from baseline to 48 weeks by paired t tests within each group, those changes were compared between the two groups an analysis of covariance (ANCOVA) after adjusting age, BMI, and baseline BMD at the L-spine. Next, BMD changes at the femur neck and TBS changes at the L-spine (secondary outcomes) were assessed by the same methods as BMD changes at the L-spine. In addition, bone turnover markers were assessed as further secondary outcomes. Occurrence of fracture and AEs was described descriptively. Statistical significance was defined as a p value < 0.05, two tailed. All analyses were performed using SAS 9.2 (SAS Institute Inc., Cary, NC, USA) and R software version 4.0.2 (R Foundation for Statistical Computing, Vienna, Austria).

## Results

### Patient enrollment and baseline characteristics

**Fig. 1:**
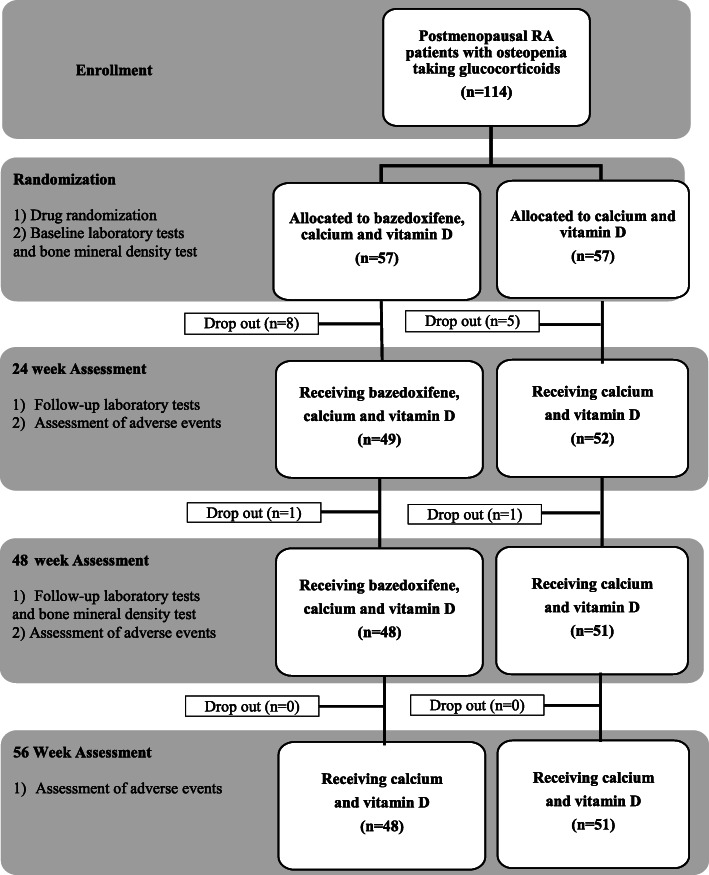
Flowchart of patient enrollment

Between December 2015 and October 2017, 114 postmenopausal women with RA were enrolled in this study and randomly assigned to either the bazedoxifene group (n = 57) or control group (n = 57). Nine participants in the bazedoxifene group withdrew at 48 weeks: three due to withdrawal of consent, five due to AEs (arthralgia, dyspnea, intervertebral disc, dyspepsia, leg pitting edema), and one due to follow-up loss. In comparison, six participants withdrew from the study in the control group: three due to AEs (one due to femur fracture, one due to vertebral fracture, one due to dyspepsia) and three due to follow-up loss. Finally, 48 participants in the bazedoxifene group and 51 participants in the control group completed the study (Fig. 1).

Mean age of the participants was 59.2 ± 6.3 years. RA disease activity was moderate with a mean DAS28-erythrocyte sedimentation rate (ESR) of 4.4 ± 1.3. Mean equivalent dose of prednisolone at baseline was 2.9 ± 1.3 mg/day. Of the total participants, 17 (14.9%) were treated with biologic disease modifying anti-rheumatic drugs (DMARDs). Baseline mean BMD, T-score, and TBS in the L-spine were 0.9 ± 0.1, − 1.3 ± 0.8, and 1.3 ± 0.1, respectively. One patient who had an L-spine compression fracture in the radiographs (grade 2 by the Genant semi-quantitative grading method) was enrolled in the bazedoxifene group. There were no significant differences in demographics or BMD between the two groups. Baseline characteristics are shown in Table 1.

**Table 1:** Baseline and clinical characteristics of recruited patients with rheumatoid arthritis (RA)

Variable	Total (n = 114)	Bazedoxifene group (n_b_ = 57)	Control group (n_c_ = 57)
Age, years	59.2 ± 6.3	59.3 ± 6.7	59.1 ± 6.0
Body mass index	23.9 ± 3.5	23.8 ± 3.9	24.0 ± 3.1
Age of menopause	48.6 ± 4.8	48.2 ± 5.0	49.1 ± 4.6
Use of hormone replacement therapy (HRT)	25 (21.9)	11 (19.3)	14 (24.6)
Duration of HRT use	22.8 ± 24.0	28.3 ± 25.3	18.6 ± 23.1
Smoking			
≤ 100/lifetime	108 (94.7)	55 (96.5)	53 (93.0)
Smoker in the past	4 (3.5)	–	4 (7.0)
Current smoker	2 (1.8)	2 (3.5)	–
Age of diagnosis with RA, years (n = 111, n_b_ = 56, n_c_ = 55)	50.5 ± 9.0	49.4 ± 9.0	51.6 ± 9.0
DAS28-ESR	4.4 ± 1.3	4.3 ± 1.3	4.4 ± 1.4
DAS28-CRP	3.3 ± 1.2	3.2 ± 1.1	3.3 ± 1.3
Comorbidities			
Charlson comorbidity index score	1.2 ± 0.5	1.3 ± 0.6	1.2 ± 0.4
Cardiovascular disease	2 (1.8)	1 (1.8)	1 (1.8)
Hypertension	42 (36.8)	24 (42.1)	18 (31.6)
Peptic ulcer disease	1 (0.9)	–	1 (1.8)
Mild liver disease	7 (6.1)	2 (3.5)	5 (8.8)
Diabetes without chronic complications	9 (7.9)	6 (10.5)	3 (5.3)
Diabetes with chronic complications	3 (2.6)	3 (5.3)	–
Laboratory tests			
Erythrocyte sedimentation rate (mm/h)	35.4 ± 26.6	34.0 ± 26.0	36.9 ± 27.5
C-reactive protein (mg/dl)	0.8 ± 1.1	0.7 ± 0.7	0.9 ± 1.3
Rheumatoid factor positivity	81 (71.1)	41 (71.9)	40 (70.2)
Anti-CCP positivity (n = 111, n_b_ = 54, n_c_ = 57)	99 (89.2)	47 (87.0)	52 (91.2)
Antinuclear antibody positivity (n = 109, n_b_ = 53, n_c_ = 56)	69 (63.3)	33 (62.3)	36 (64.3)
Bone-specific alkaline phosphatase	14.7 ± 4.7	14.3 ± 4.2	15.1 ± 5.1
Osteocalcin	19.8 ± 9.5	20.5 ± 10.6	19.1 ± 8.2
C-telopeptide	0.5 ± 0.2	0.5 ± 0.2	0.5 ± 0.2
N-telopeptide	46.5 ± 18.5	46.2 ± 19.6	46.7 ± 17.6
Bone mineral density			
Lumbar spine	0.9 ± 0.1	0.9 ± 0.1	0.9 ± 0.1
Lt. hip neck	0.6 ± 0.1	0.6 ± 0.1	0.6 ± 0.1
Rt. hip neck	0.6 ± 0.1	0.6 ± 0.1	0.6 ± 0.1
T-score			
L-spine	− 1.3 ± 0.8	− 1.3 ± 0.8	− 1.3 ± 0.7
Lt. hip neck	− 1.9 ± 1.9	− 1.7 ± 0.7	− 2.0 ± 2.7
Rt. hip neck	− 1.6 ± 0.7	− 1.6 ± 0.6	− 1.5 ± 0.8
Trabecular bone score	1.3 ± 0.1	1.3 ± 0.1	1.3 ± 0.1
Fracture risk assessment tool (FRAX) score	13.8 ± 5.1	14.6 ± 5.0	13.0 ± 5.1
Hip FRAX score	5.3 ± 3.7	5.7 ± 3.5	4.9 ± 3.8
Patients at high risk of fracture^*^	80 (70.2)	40 (70.2)	40 (70.2)
Patients at moderate or high risk of fracture^†^	112 (98.2)	56 (98.2)	56 (98.2)
Current medications			
DMARDs	114 (100.0)	57 (100.0)	57 (100.0)
Methotrexate	95 (83.3)	50 (87.7)	45 (78.9)
Biologic DMARDs	17 (14.9)	9 (15.8)	8 (14.0)
Etanercept	7 (6.1)	4 (7.0)	3 (5.3)
Abatacept	5 (4.4)	4 (7.0)	1 (1.8)
Golimumab	3 (2.6)	1 (1.8)	2 (3.5)
Adalimumab	2 (1.8)	–	2 (3.5)
Non-selective NSAIDs	11 (9.6)	5 (8.8)	6 (10.5)
COX2 selective inhibitor	85 (74.6)	43 (75.4)	42 (73.7)
Equivalent dose of prednisolone (mg/day)^‡^ (*n* = 107, *n*_*b*_ = 53, *n*_*c*_ = 54)	3.0 (1.1–10.0)	3.2 (1.1–10.0)	2.7 (1.3–5.0)
HAQ	0.6 ± 0.6	0.7 ± 0.6	0.5 ± 0.5
EuroQol-5 dimension	0.8 ± 0.1	0.8 ± 0.1	0.8 ± 0.1
Visual analog scale (VAS) of pain	43.2 ± 27.2	44.4 ± 26.4	41.9 ± 28.2
VAS of global health	41.3 ± 25.7	41.9 ± 25.4	40.7 ± 26.3
VAS of sleep	28.1 ± 28.7	28.1 ± 28.7	28.1 ± 29.0
VAS of fatigue	40.3 ± 26.8	42.6 ± 27.2	37.9 ± 26.4

Continuous data are presented as mean ± SD, and categorical data are presented as frequency (%)

^*^High risk of fracture was defined as a 10-year-probability of major osteoporotic fracture exceeding 20% or a 10-year probability of hip fracture exceeding 3% based on the fracture risk assessment tool (FRAX)

^†^Moderate or high risk of fracture was defined as a 10-year-probability of major osteoporotic fracture exceeding 10% or a 10-year probability of hip fracture exceeding 1% based on the FRAX tool

^‡^Dose of glucocorticoid is presented as mean ± SD (range)

DAS, disease activity score, ESR, erythrocyte sedimentation rate, CRP, C-reactive protein, Anti-CCP, anti-cyclic citrullinated peptide antibody, DMARDs, disease-modifying antirheumatic drugs, NSAIDs, nonsteroidal anti-inflammatory drugs, HAQ, health assessment questionnaire-disability index

### Primary outcome: changes in L-spine BMD

**Fig. 2:**
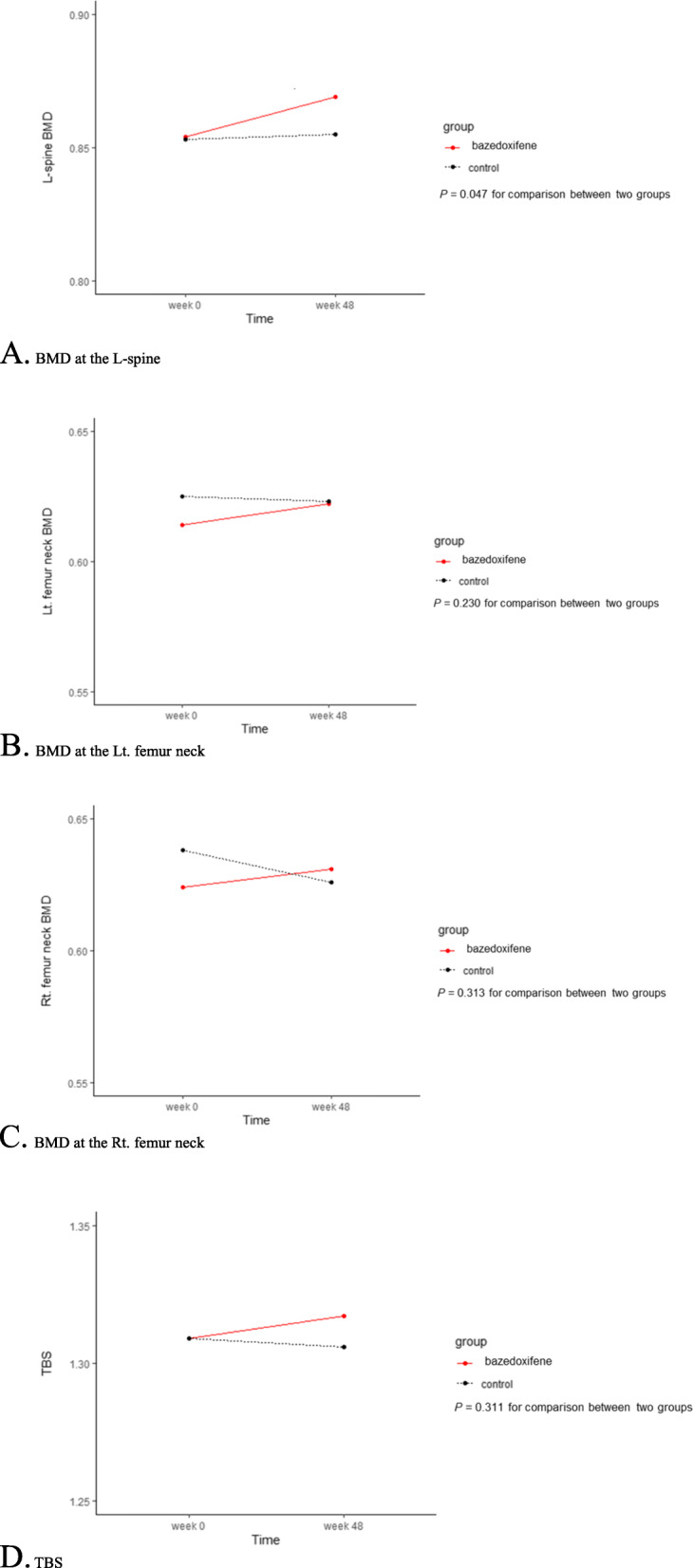
Changes in BMD and TBS from baseline to 12 months in the total patients (n = 114). BMD, bone mineral density, TBS, trabecular bone score. **A** BMD at the L-spine. **B** BMD at the Lt. femur neck. **C** BMD at the Rt. femur neck. **D** TBS

In the primary analysis, BMD change at the L-spine was assessed in an intend-to-treat population of 114 patients after imputing missing observations. Figure 2A and Additional file 2 show the changes in L-spine BMD from baseline at 48 weeks in the two groups. A significant gain in L-spine BMD compared to baseline was observed in the bazedoxifene group (0.015 g/cm^2^ from 0.854 to 0.869, SE = 0.005, *P* = 0.007), while there was no significant change in L-spine BMD in the control group (0.002 g/cm^2^ from 0.853 to 0.855, 0.2%, SE = 0.004, *P* = 0.694). A significant difference in the change of L-spine BMD between the two groups persisted after adjusting age, BMI, and baseline BMD at the L-spine (0.013, 95% CI 0.0003-0.026, *P* = 0.047).

Per-protocol analysis (Additional file 3) with 99 participants (48 in the bazedoxifene group and 51 in the control group) completed the study as the protocol is performed. In this population, BMD gain at the L-spine was also significant in the bazedoxifene group (0.014 g/cm^2^ from 0.861 to 0.875, SE = 0.005, *P* = 0.012), while there was no significant change in the control group (0.001 g/cm^2^ from 0.859 to 0.860, SE = 0.005, *P* = 0.865). A significant difference in the change of L-spine BMD was maintained between the two groups after adjusting age, BMI, and baseline BMD at the L-spine (*P* = 0.048). Thus, the result from the per-protocol analysis was consistent with the intend-to-treat analysis.

A subgroup analysis was performed in a group of patients at high risk of fracture (Additional file 4). These patients at high risk of fracture were identified as having a 10-year major osteoporotic fracture probability exceeding 20% or hip fracture exceeding 3%. Eighty patients (45 in the bazedoxifene group, and 35 in the control group) were included in this analysis. Again, BMD gain at the L-spine was significant in the bazedoxifene group (0.020 g/cm^2^ from 0.850 to 0.870, SE = 0.006, *P* = 0.002), but not in the control group (0.008 g/cm^2^ from 0.855 to 0.863, SE = 0.006, *P* = 0.166). However, the changes in L-spine BMD from baseline to 48 weeks were not significantly different between the two groups after adjusting age, BMI, and baseline L-spine BMD (*P* = 0.094).

### Secondary outcomes

#### Changes in femur neck BMD

The BMD of the femur neck did not change either in the bazedoxifene group (0.008 g/cm^2^ from 0.614 to 0.622, SE = 0.007, *P* = 0.078 for the left, and 0.007 g/cm^2^ from 0.624 to 0.631, SE = 0.007, *P* = 0.315 for the right) or the control group (− 0.003 g/cm^2^ from 0.625 to 0.622, SE = 0.006, *P* = 0.671 for the left and − 0.012 g/cm^2^ from 0.638 to 0.626, SE = 0.012, *P* = 0.347 for the right). The changes in left femur neck BMD from baseline to 48 weeks in the two groups were not significantly after adjusting age, BMI, and baseline left femur neck BMD (*P* = 0.230), and the same was true for the changes in right femur neck BMD (*P* = 0.313) (Fig. 2B: left femur neck, Fig. 2C: right femur neck, Additional file 2).

#### Changes in TBS

The changes in TBS at the L-spine from baseline to 48 weeks did not differ significantly different either in the bazedoxifene group (0.008 from 1.309 to 1.317, SE = 0.006, *P* = 0.202) or the control group (− 0.003 from 1.309 to 1.306, SE = 0.010, *P* = 0.794) group (Fig. 2D). The changes in the TBS were not significantly different between the two groups after adjusting age, BMI, and baseline TBS (*P* = 0.311) (Additional file 2).

#### Changes in markers of bone turnover

**Fig. 3:**
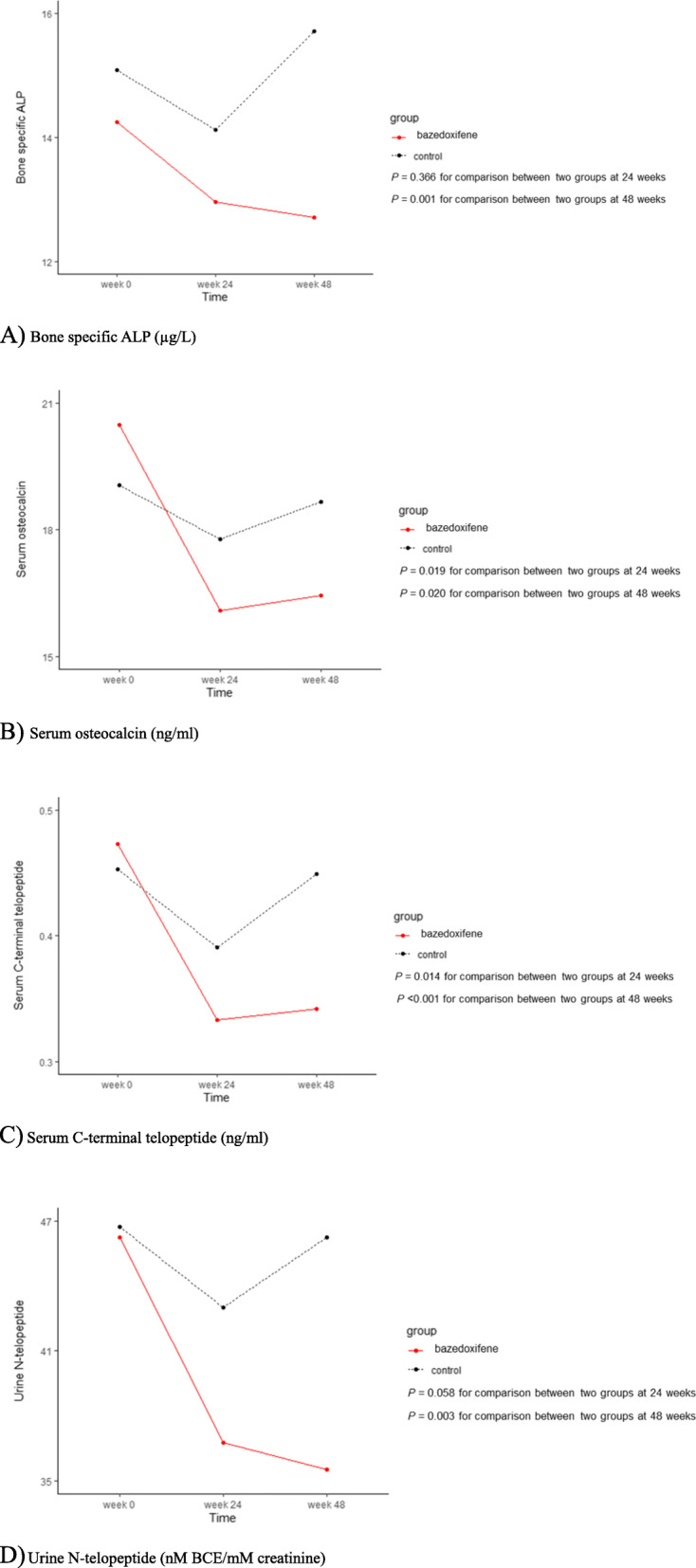
Changes in bone turnover markers from baseline to 6 and 12 months in the total patients (n = 114). ALP, alkaline phosphatase. **A** Bone-specific ALP (μg/L). **B** Serum osteocalcin (ng/ml). **C** Serum C-terminal telopeptide (ng/ml). **D** Urine N-telopeptide (nM BCE/mM creatinine)

In the bazedoxifene group, significant decreases in all bone turnover biomarkers were observed at 24 weeks and persisted to 48 weeks (all *P* < 0.01). In contrast, the decrease in bone markers recovered by 48 weeks in the control group, although the differences were not significant. The reductions in serum levels of bone-specific ALP and urine N-telopeptide were significantly greater at 48 weeks in the bazedoxifene group than in the control group after adjusting age, BMI, and baseline serum levels of bone-specific ALP values for the corresponding bone markers (*P* = 0.001, and 0.003), even though the reductions at 24 weeks in the two groups were not significantly different (Figs. 3A and 3D). On the other hand, reductions in bone formation marker (serum osteocalcin) and resorption marker (serum C-telopeptide) were significantly greater at both 24 and 48 weeks in the bazedoxifene group than the control group after adjustment (*P* = 0.019, and 0.014 at 24 weeks, and *P* = 0.020, and < 0.001 at 48 weeks) (Figs. 3B and 3C, Additional file 5).

#### Occurrence of fractures

Fractures occurred in five patients: one rib fracture in a patient in the bazedoxifene group, and four fractures in the control group (two hip, one spine, and one wrist).

### Adverse events

More AEs were reported in the bazedoxifene group (46 of 57 patients [80.7%]) than in the control group (23 of 57 patients [40.4%]) (Table 2). However, serious adverse events (SAEs) were more frequent in the control group (10 of 57 patients [17.5%]) than in the bazedoxifene group (7 of 57 patients [12.3%]). Specifically, ten gastrointestinal disorders (17.5%), including dyspepsia [4] and constipation [2], were the most common AE in the bazedoxifene group. Injury was frequent in the control group (1.8% in the bazedoxifene group vs. 5.3% in the control group), while infection was more frequent in the bazedoxifene group (5.3% vs. 1.8%). Seven cases of infection (12.2%), namely vaginitis (n = 2), herpes zoster (n = 2), periodontitis (n = 1), acute pyelonephritis (n = 1), and pneumonia (n = 1) were reported in the bazedoxifene group, while one case of infective bursitis was reported in the control group.

**Table 2:** Adverse events in patients with RA (n = 114)

Systemic organ	Bazedoxifene group (n = 57)	Control group (n = 57)
Adverse events (AE): no. of patients (%)	27 (47.4)	15 (26.3)
Musculoskeletal	8 (14.0)^§^	4 (7.0)^§^
Gastrointestinal disorder	8 (14.0)^¶^	2 (3.5)^§^
Infection^*^	5 (8.8)^¶^	1 (1.8)
Respiratory	2 (3.5)	4 (7.0)
Injury^†^	2 (3.5)	3 (5.3)
Hepatobiliary disorder	3 (5.3)^¶^	1 (1.8)
Skin and subcutaneous tissue disorder	2 (3.5)	2 (3.5)
General disorder	3 (5.3)	0 (0.0)
Hot flushing	2 (3.5)	0 (0.0)
Nervous system disorder	2 (3.5)	0 (0.0)
Renal and urinary	1 (1.8)	1 (1.8)
Other^‡^	1 (1.8)	3 (5.3)
Total number of AE	46	23
Serious adverse events (SAE): no. of patients (%)	7 (12.3)	10 (17.5)
Musculoskeletal	3 (5.3)	3 (5.3)
Infection^£^	3 (5.3)	1 (1.8)
Injury^€^	1 (1.8)	3 (5.3)
Renal and urinary	0	1 (1.8)
Eye	0	1 (1.8)
Malignancy	0	1 (1.8)
Total number of SAE	7	10

^*^Infection includes one patient with two skin and soft tissues infections, one patient with two reproductive organ infection, one pulmonary, one urinary, and one gum infection in the bazedoxifene group, and one skin and soft tissue infection in the control group

^†^All injuries, except for one skin abrasion in the bazedoxifene group, were fractures, including one rib fracture in the bazedoxifene group, two hips, one spine, and one wrist fracture in the control group

^‡^Other includes one psychiatric episode in the bazedoxifene group, one cardiac, one eye disorder, and one malignancy in the control group

^£^Infections include one pulmonary, one skin and soft tissue, and one urinary infection in the bazedoxifene group, one skin and soft tissue infection in the control group

^€^All injuries were fractures with one rib fracture in the bazedoxifene group, two hip fractures, and one spine fracture in the control group

^§^One patient with two AEs at different times or sites, ^¶^One patient with three AS at different times or sites

## Discussion

There was a significant increase (0.015 g/cm^2^, *P* = 0.0071) in the BMD of the L-spine of RA patients after 48 weeks of treatment with bazedoxifene, while no significant change was observed in the control group. A significant difference in the change of L-spine BMD between the two groups persisted after adjusting age, BMI, and baseline BMD at the L-spine. However, the change of BMD by bazedoxifene did not exceed the LSC. Bazedoxifene significantly reduced serum levels of bone formation markers (serum bone-specific ALP and osteocalcin) and resorption markers (serum C-telopeptide and urine N-telopeptide) compared to patients who did not take bazedoxifene.

A previous 3-year RCT of patients with osteoporosis found that bazedoxifene 20 mg significantly increased BMD in the spine and hip (2.2% and 0.3%, respectively) compared to placebo (0.9% and − 0.8%) [20]. Bone turnover markers in patients with osteoporosis were also reduced after 12 months of treatment (osteocalcin by 37% and CTX by 46%) [20]. In the current study, the percentage increase in BMD (1.8% in the L-spine and 1.3% in the left femur neck) and bone markers (12–38%) after treatment with bazedoxifene for 12 months was lower than those reported in the previous RCT. This smaller change in BMD after treatment with bazedoxifene may be responsible for the nonsignificant difference in BMD between the bazedoxifene group and control group; we attribute this to differences between our study population and to the study population analyzed in the previous RCT. To elaborate, we enrolled RA patients with osteopenia and excluded patients already diagnosed with osteoporosis to generate evidence regarding bazedoxifene treatment for patients who do not meet the criteria of osteoporosis but are at risk for GIOP. Our definition of osteopenia as a T score between − 1.0 and − 2.5 resulted in the inclusion of patients with mild osteopenia who had little room for improvement in their BMD. Another reason for the smaller changes in BMD and bone markers after bazedoxifene treatment in our study could be our inclusion of patients treated with GCs for more than 3 months prior to entry. Previous longitudinal studies have suggested that bone loss may be more rapid soon after starting treatment with GCs, after which it decreases slowly [21]. Thus, the BMD of our population receiving GC treatment may not have decreased rapidly during our study period. Furthermore, the mean doses of glucocorticoids decreased over the study period, although there was no significant difference between two groups. The extremely low-dose GCs in our study may have led to failure to demonstrate a benefit of bazedoxifene compared with the control group. These factors should be taken into account in future studies when considering sample sizes. A subgroup analysis in patients at high risk of fracture based on FRAX failed to reveal a significant difference between the bazedoxifene and control groups, even though bazedoxifene improved BMD at 48 weeks. This lack of statistical significance may be due to our small sample size.

Nevertheless, our study demonstrated that only one fracture developed in the bazedoxifene group compared with four in the control group. Major osteoporotic fractures such as vertebral and hip fractures developed exclusively in the patients not treated with bazedoxifene. Furthermore, the significant increase in BMD after treatment with bazedoxifene suggests that bazedoxifene helps to reduce early bone loss in patients who use low-dose GCs.

In terms of the safety of bazedoxifene, the most common AE in the bazedoxifene group was dyspepsia, consistent with the report of development of abdominal pain in a 7-year randomized, placebo-controlled trial of bazedoxifene in patients with osteoporosis [14]. Although there was a higher incidence of infection in the bazedoxifene group than in the control group, the relationship between bazedoxifene and the development of infection is unclear, and all cases showed substantial or complete recovery. No venous thromboembolism was found in this study, which is consistent with the recent post-marketing surveillance study [22]. A further long-term safety study is needed to examine this issue.

The prevalence of osteoporosis in patients with RA is 30% and increases to 50% in postmenopausal patients [17], and the fracture risk is twofold greater in patients with RA than those without RA [23]. The number of patients with RA who receive GC therapy to control disease activity is substantial [24, 25]. Nevertheless, the prevention and management of osteoporosis in postmenopausal patients with RA are inadequate [26, 27]. Bisphosphonates are frequently used to treat this population [26], and postmenopausal patients with a long-term history of RA are likely long-term users of bisphosphonates. Sequential treatment with several osteoporosis medications is crucial to prevent osteopenia progressing to osteoporosis. Further study with a larger sample size and longer observational period is required to confirm the potential effectiveness of bazedoxifene to prevent bone loss with GC in postmenopausal patients with RA. However, in order to prescribe this drug as a stand-alone, it is important to evaluate the cardiovascular risks of long-term use. In addition, there are differences in the availability of this drug across countries. In the USA, bazedoxifene is only approved as a combination drug with a conjugate estrogen, and separate clinical trials may be required for its use as a stand-alone. Hence, these constraints in the use of bazedoxifene will need to be resolved before it can be used in postmenopausal women with osteopenia or osteoporosis using GCs.

The limitations of our study included its open-label design, which may have affected drug compliance or reporting patients’ reported outcomes. However, drug compliance was similar in the two groups ( Additional file 6), and the outcomes of our study were objective indicators such as changes of BMD, TBS, and bone turnover markers, and the spine x-rays were evaluated by blinded radiologists. Nevertheless, an open-label study design may have led to a difference in glucocorticoid maintenance over the study period as there may have been a limited effort to lower GC in the bazedoxifene group. Our study design including patients with osteopenia using low-dose GC in addition to its relatively short observational period may have reduced the effect size. It could also have been responsible for the low statistical power. These factors should be taken into account in future studies when considering sample sizes. Finally, our study has low statistical power: the comparison of the changes in L-spine BMD between the two groups is supported by only 50.4% power, whereas the comparison of the L-spine BMD between baseline and 48 weeks is supported by 83.8% power in the bazedoxifene group. Long-term clinical trials with sufficient sample sizes to assess if bazedoxifene can prevent fractures in patients with GIOP are needed.

## Conclusion

We found a significant increase in the BMD of the L-spine of postmenopausal women with RA receiving low-dose GCs after treatment with bazedoxifene for 48 weeks. A significant difference in the change of L-spine BMD between the two groups was found. However, the change in BMD by bazedoxifene did not exceed the LSC*.*

## Supplementary Information

**Additional file 1.** Study design.

**Additional file 2.** Change in BMD and TBS from baseline to 12 months in the total patient: within- and between-group comparisons (n = 114).

**Additional file 3.** Change in BMD and TBS from baseline to 12 months in the per-protocol population: within- and between-group comparisons (n = 99).

**Additional file 4.** Change in BMD and TBS from baseline to 12 months in the high-risk patients of fracture: within- and between-group comparisons (n = 80).

**Additional file 5.** Changes in bone turnover markers from baseline to 12 in the total patient: within- and between-group comparisons (n = 114).

**Additional file 6.** Drug compliance (%).

## Data Availability

The datasets used and/or analyzed during the current study are available from the corresponding author on reasonable request.

## Abbreviations

RA: Rheumatoid arthritis
GCs: Glucocorticoids
BMD: Bone mineral density
TBS: Trabecular bone score
IL: Interleukin
TNF-α: Tumor necrosis factor-α
GIOP: Glucocorticoid-induced osteoporosis
FRAX: Fracture risk assessment tool
AEs: Adverse events
SERMs: Selective estrogen receptor modulators
RCT: Randomized controlled trial
CBC: Complete blood count
DXA: Dual-energy x-ray absorptiometry
ALP: Bone-specific alkaline phosphatase
SD: Standard deviations
SE: Standard error
MICE: Multiple imputations by chained equations
CART: Classification and regression tree
ANOVA: Analysis of variance
ANCOVA: Analysis of covariance
ESR: Erythrocyte sedimentation rate
DMARDs: Disease modifying anti-rheumatic drugs
SAEs: Serious adverse events
