# Supplementary material for: Effectiveness of bazedoxifene in preventing glucocorticoid-induced bone loss in rheumatoid arthritis patients
Source: Arthritis Res Ther. 2021 Jul 2;23:176. doi: 10.1186/s13075-021-02564-1 (PMC8252248; doi:10.1186/s13075-021-02564-1)
Supplement: Supplementary file 1 — Additional file 1. Study design. [file 13075_2021_2564_MOESM1_ESM.docx]

Additional file 1. Study design.

Visit 0

(Screening)

Visit 1

(0 week)

Visit 2

(24 weeks)

Visit 3

(48 weeks)

Visit 4

(56 weeks)

Bazedoxifene group

(elemental calcium 1200 mg/day and vitamin D 800 IU/day + bazedoxifene 20 mg/day)

Control group

(elemental calcium 1200 mg/day and vitamin D 800 IU/day)

Screening

Randomization

Outcome Measurement

Physical examination

DXA

TBS

Bone turnover markers

RA disease activity

Physical examination

Bone turnover markers

RA disease activity

Safety assessment

Physical examination

DXA

TBS

Bone turnover markers

RA disease activity

Safety assessment

Safety assessment
