# Supplementary material for: Effectiveness of bazedoxifene in preventing glucocorticoid-induced bone loss in rheumatoid arthritis patients
Source: Arthritis Res Ther. 2021 Jul 2;23:176. doi: 10.1186/s13075-021-02564-1 (PMC8252248; doi:10.1186/s13075-021-02564-1)
Supplement: Supplementary file 2 — Additional file 2. Change in BMD and TBS from baseline to 12 months in the total patient: within- and between-group comparisons (n = 114). [file 13075_2021_2564_MOESM2_ESM.docx]

Additional file 2. Changes in BMD and TBS from baseline to 12 months in the total patients: within- and between-group comparisons (n = 114)^*^

|  | Bazedoxifene group (n = 57) | | | | | Control group (n = 57) | | | | | Comparison between group (n = 114) | | |
| --- | --- | --- | --- | --- | --- | --- | --- | --- | --- | --- | --- | --- | --- |
|  | Week 0 | Week 48 | Change | SE | *P* ^†^ | Week 0 | Week 48 | Change | SE | *P* ^†^ | Difference | 95% C.I. | *P* ^‡^ |
| L-spine BMD | 0.854 | 0.869 | 0.015 | 0.005 | 0.007 | 0.853 | 0.855 | 0.002 | 0.004 | 0.734 | 0.013 | ( 0.0003, 0.026) | 0.047 |
| Lt. femur neck BMD | 0.614 | 0.621 | 0.007 | 0.005 | 0.119 | 0.625 | 0.623 | -0.003 | 0.006 | 0.642 | 0.009 | (-0.006, 0.024) | 0.256 |
| Rt. femur neck BMD | 0.624 | 0.631 | 0.006 | 0.006 | 0.268 | 0.638 | 0.625 | -0.013 | 0.012 | 0.291 | 0.016 | (-0.010, 0.042) | 0.239 |
| TBS | 1.309 | 1.317 | 0.008 | 0.006 | 0.192 | 1.309 | 1.306 | -0.003 | 0.010 | 0.777 | 0.011 | (-0.010, 0.031) | 0.289 |

BMD: bone mineral density, TBS: trabecular bone score, SE: standard error

^*^ This result is from an intention-to-treat analysis with multiple imputations by chained equations (MICE)

^†^ BMD and TBS at 48 weeks are compared to the baseline by paired t-tests in each group (within-group analysis).

**^‡^** Changes in BMD and TBS were compared between the two groups by analyses of covariance (ANCOVA) after adjusting age, body mass index, and the baseline value of the corresponding outcome (between-group analysis).
