# Supplementary material for: Effectiveness of bazedoxifene in preventing glucocorticoid-induced bone loss in rheumatoid arthritis patients
Source: Arthritis Res Ther. 2021 Jul 2;23:176. doi: 10.1186/s13075-021-02564-1 (PMC8252248; doi:10.1186/s13075-021-02564-1)
Supplement: Supplementary file 3 — Additional file 3. Change in BMD and TBS from baseline to 12 months in the per-protocol population: within- and between-group comparisons (n = 99). [file 13075_2021_2564_MOESM3_ESM.docx]

Additional file 3. Changes in BMD and TBS from baseline to 12 months in the per-protocol population: within- and between-group comparisons (n = 99)

|  | Bazedoxifene group (n = 48) | | | | | Control group (n = 51) | | | | | Comparison between group (n = 99) | | |
| --- | --- | --- | --- | --- | --- | --- | --- | --- | --- | --- | --- | --- | --- |
|  | Week 0 | Week 48 | Change | SE | *P* ^†^ | Week 0 | Week 48 | Change | SE | *P* ^†^ | Difference | 95% C.I. | *P* ^‡^ |
| L-spine BMD | 0.861 | 0.875 | 0.014 | 0.005 | 0.012 | 0.859 | 0.860 | 0.001 | 0.005 | 0.865 | 0.013 | (0.0003, 0.027) | 0.048 |
| Lt. femur neck BMD | 0.618 | 0.627 | 0.009 | 0.005 | 0.058 | 0.631 | 0.627 | -0.005 | 0.007 | 0.506 | 0.006 | (-0.002, 0.014) | 0.138 |
| Rt. femur neck BMD | 0.625 | 0.631 | 0.006 | 0.006 | 0.363 | 0.646 | 0.624 | -0.022 | 0.012 | 0.080 | 0.012 | (-0.002, 0.026) | 0.095 |
| TBS | 1.314 | 1.320 | 0.007 | 0.006 | 0.258 | 1.307 | 1.305 | -0.001 | 0.010 | 0.912 | 0.008 | (-0.005, 0.016) | 0.298 |

BMD: bone mineral density, TBS: trabecular bone score, SE: standard error

^†^ BMD and TBS at 48 weeks are compared to baseline by paired t-tests in each group (within-group analysis).

**^‡^** Changes in BMD and TBS were compared between the two groups by analyses of covariance (ANCOVA) after adjusting age, BMI, and baseline value of the corresponding outcome (between-group analysis).
