# Supplementary material for: Effectiveness of bazedoxifene in preventing glucocorticoid-induced bone loss in rheumatoid arthritis patients
Source: Arthritis Res Ther. 2021 Jul 2;23:176. doi: 10.1186/s13075-021-02564-1 (PMC8252248; doi:10.1186/s13075-021-02564-1)
Supplement: Supplementary file 4 — Additional file 4. Change in BMD and TBS from baseline to 12 months in the high-risk patients of fracture: within- and between-group comparisons (n = 80). [file 13075_2021_2564_MOESM4_ESM.docx]

Additional file 4. Changes in BMD and TBS from baseline to 12 months in the high-risk patients of fracture: within- and between-group comparisons (n = 80)

|  | Bazedoxifene group (n = 40) | | | | | Control group (n = 40) | | | | | Comparison between group (n = 80) | | |
| --- | --- | --- | --- | --- | --- | --- | --- | --- | --- | --- | --- | --- | --- |
|  | Week 0 | Week 48 | Change | SE | *P* ^†^ | Week 0 | Week 48 | Change | SE | *P* ^†^ | Difference | 95% C.I. | *P* ^‡^ |
| L-spine BMD | 0.850 | 0.870 | 0.020 | 0.006 | 0.002 | 0.855 | 0.863 | 0.008 | 0.006 | 0.166 | 0.014 | (-0.002, 0.029) | 0.094 |
| Lt. femur neck BMD | 0.596 | 0.610 | 0.015 | 0.005 | 0.011 | 0.601 | 0.605 | 0.004 | 0.006 | 0.547 | 0.011 | (-0.005, 0.027) | 0.166 |
| Rt. femur neck BMD | 0.614 | 0.621 | 0.007 | 0.008 | 0.377 | 0.613 | 0.617 | 0.003 | 0.008 | 0.665 | 0.004 | (-0.016, 0.023) | 0.711 |
| TBS | 1.298 | 1.313 | 0.015 | 0.006 | 0.009 | 1.299 | 1.299 | 0.000 | 0.013 | 0.975 | 0.011 | (-0.011, 0.033) | 0.346 |

BMD: bone mineral density, TBS: trabecular bone score, SE: standard error

The high-risk group was defined by patients with a 10-year-major osteoporotic fracture probability exceeding 20% or hip fracture probability exceeding 3% based on the fracture risk assessment tool (FRAX).

^†^ BMD and TBS at 48 weeks were compared to the baseline by paired t-tests in each group (within-group analysis).

**^‡^** Changes in BMD and TBS were compared between two groups using analyses of covariance (ANCOVA) after adjusting age, BMI, and baseline value of the corresponding outcome (between-group analysis)
