# Supplementary material for: Effectiveness of bazedoxifene in preventing glucocorticoid-induced bone loss in rheumatoid arthritis patients
Source: Arthritis Res Ther. 2021 Jul 2;23:176. doi: 10.1186/s13075-021-02564-1 (PMC8252248; doi:10.1186/s13075-021-02564-1)
Supplement: Supplementary file 5 — Additional file 5. Changes in bone turnover markers from baseline to 12 in the total patient: within- and between-group comparisons (n = 114). [file 13075_2021_2564_MOESM5_ESM.docx]

Additional file 5. Changes in bone turnover markers from baseline to 12 months in the total patients: within- and between-group comparisons (n = 114)

|  | Bazedoxifene group (n = 57) | | | | | Control group (n = 57) | | | | | Comparison between group (n = 114) | | |
| --- | --- | --- | --- | --- | --- | --- | --- | --- | --- | --- | --- | --- | --- |
|  | Mean | SE | Change | SE | *P* ^†^ | Mean | SE | Change | SE | *P* ^†^ | Difference | 95% C.I. | *P* ^‡^ |
| Bone specific ALP |  |  |  |  |  |  |  |  |  |  |  |  |  |
| Baseline | 14.254 | 0.563 |  |  |  | 15.084 | 0.674 |  |  |  |  |  |  |
| Week 24 | 12.956 | 0.604 | -1.299 | 0.428 | 0.004 | 14.126 | 0.629 | -0.958 | 0.521 | 0.072 | -0.558 | (-1.763, 0.646) | 0.366 |
| Week 48 | 12.711 | 0.487 | -1.544 | 0.462 | 0.002 | 15.709 | 0.790 | 0.625 | 0.587 | 0.292 | -2.389 | (-3.759, -1.019) | 0.001 |
| Serum osteocalcin |  |  |  |  |  |  |  |  |  |  |  |  |  |
| Baseline | 20.488 | 1.409 |  |  |  | 19.051 | 1.085 |  |  |  |  |  |  |
| Week 24 | 16.081 | 1.071 | -4.407 | 0.930 | < 0.001 | 17.775 | 0.956 | -1.276 | 0.905 | 0.165 | -2.474 | (-4.512, -0.436) | 0.019 |
| Week 48 | 16.438 | 1.135 | -4.050 | 1.069 | < 0.001 | 18.666 | 1.029 | -0.384 | 1.012 | 0.706 | -2.868 | (-5.232, -0.505) | 0.020 |
| C- telopeptide |  |  |  |  |  |  |  |  |  |  |  |  |  |
| Baseline | 0.473 | 0.028 |  |  |  | 0.453 | 0.023 |  |  |  |  |  |  |
| Week 24 | 0.333 | 0.025 | -0.140 | 0.021 | < 0.001 | 0.391 | 0.026 | -0.062 | 0.023 | 0.009 | -0.070 | (-0.125, -0.015) | 0.014 |
| Week 48 | 0.342 | 0.030 | -0.131 | 0.022 | < 0.001 | 0.449 | 0.031 | -0.004 | 0.024 | 0.858 | -0.121 | (-0.184, -0.058) | < 0.001 |
| N-telopeptide |  |  |  |  |  |  |  |  |  |  |  |  |  |
| Baseline | 46.246 | 2.590 |  |  |  | 46.737 | 2.329 |  |  |  |  |  |  |
| Week 24 | 36.749 | 2.474 | -9.496 | 2.361 | < 0.001 | 43.020 | 2.604 | -3.717 | 2.559 | 0.152 | -5.909 | (-11.948, 0.131) | 0.058 |
| Week 48 | 35.508 | 2.317 | -10.738 | 2.761 | < 0.001 | 46.239 | 2.988 | -0.497 | 2.853 | 0.862 | -10.374 | (-17.142, -3.607) | 0.003 |

ALP: alkaline phosphatase, SE: standard error

^†^ Bone turnover markers in each follow-up visit were compared to the baseline values by paired t-tests in each group (within-group analysis).

^‡^ Changes in bone turnover markers at 24 and 48 weeks were compared between the two groups by analyses of covariance (ANCOVA) after adjusting age, BMI, and the baseline value of the corresponding bone marker (between-group analysis).
