# Supplementary material for: Effectiveness of bazedoxifene in preventing glucocorticoid-induced bone loss in rheumatoid arthritis patients
Source: Arthritis Res Ther. 2021 Jul 2;23:176. doi: 10.1186/s13075-021-02564-1 (PMC8252248; doi:10.1186/s13075-021-02564-1)
Supplement: Supplementary file 6 — Additional file 6. Drug compliance (%). [file 13075_2021_2564_MOESM6_ESM.docx]

Additional file 6. Drug compliance (%)

|  |  | Bazedoxifene group | Control group | p-value |
| --- | --- | --- | --- | --- |
| Bazedoxifene | 24 weeks | 91.95 | - | - |
|  | 48 weeks | 90.69 | - | - |
| Calcium  and vitamin D | 24 weeks | 84.32 | 89.72 | 0.117 |
|  | 48 weeks | 83.58 | 86.25 | 0.479 |
